# Addictive brain-network identification by spatial attention recurrent network with feature selection

**DOI:** 10.1186/s40708-022-00182-4

**Published:** 2023-01-10

**Authors:** Changwei Gong, Xinyi Chen, Bushra Mughal, Shuqiang Wang

**Affiliations:** 1 Shenzhen Institutes of Advanced Technology, Chinese Academy of Sciences, Shenzhen, 518055 China; 2 University of Chinese Academy of Sciences, Beijing, 100049 China; 3 Southern University of Science and Technology, Shenzhen, 518055 China; 4 Faculty of Engineering, University of Porto, Porto, 0035122 Portugal

**Keywords:** Neural imaging computing, Brain network, Graph neural networks

## Abstract

Addiction in the brain is associated with adaptive changes that reshape addiction-related brain regions and lead to functional abnormalities that cause a range of behavioral changes, and functional magnetic resonance imaging (fMRI) studies can reveal complex dynamic patterns of brain functional change. However, it is still a challenge to identify functional brain networks and discover region-level biomarkers between nicotine addiction (NA) and healthy control (HC) groups. To tackle it, we transform the fMRI of the rat brain into a network with biological attributes and propose a novel feature-selected framework to extract and select the features of addictive brain regions and identify these graph-level networks. In this framework, spatial attention recurrent network (SARN) is designed to capture the features with spatial and time-sequential information. And the Bayesian feature selection(BFS) strategy is adopted to optimize the model and improve classification tasks by restricting features. Our experiments on the addiction brain imaging dataset obtain superior identification performance and interpretable biomarkers associated with addiction-relevant brain regions.

## Introduction

Neuroscience is stepping into a period marked by large amounts of complex neural data obtained from large-scale neural systems [1]. Most of these large data are presented in the form of data from networks covering the relationships or interconnections of elements within different types of large-scale neurobiological systems, for example, connections and anatomical projections of neural circuitry between brain regions and patterns of neural signals in brain regions associated with spontaneous and task-induced brain activities. Brain networks are segmented by anatomical structures that partition different brain regions and connect them together, and functional brain networks display complex neuronal communication and signaling patterns.

Moreover, neuroimaging [2] is a bridging field that integrates medical imaging computing and neuroscience and has been evolving in recent years. Brain imaging [3] is a powerful tool for studying neuroscience, diagnosing and treating brain disorders through qualitative and quantitative analysis of two- and three-dimensional images [4], and using imaging methods to explain the anatomical structure and activity of the brain [5], as well as to address unanswered questions in the field of neuroscience [6, 7]. Addiction is a brain dysfunction characterized by abnormal behavior, and addicts are driven by an overwhelming compulsion to seek and consume drugs constantly. Drug addiction treatment is difficult [8], and its biological mechanisms have not been fully illuminated. Meanwhile, imaging studies have revealed neurochemical and functional changes in the brains of addicted individuals, providing new insights into the mechanisms of addiction.

Owing to advances in modern imaging techniques and advanced medical image analysis methods [9], patterns of such complex neural signals can be analyzed from functional images, which reveal their association with neuronal activity [10], such as behavior and cognition, as well as brain diseases [11]. However, few computational brain imaging methods use functional MRI to investigate the relationship between nicotine addiction and altered neuronal activity patterns throughout the brain [12], identify these patterns and detect regional neuroimaging biomarkers. Therefore, brain imaging studies of the neural mechanisms and supporting diagnoses associated with nicotine and other drug addiction have become increasingly critical.

Functional magnetic resonance (fMRI) [13] has been used to study nicotine dependence’s neural basis and develop smoking cessation strategies. Resting-state functional magnetic resonance imaging (rs-fMR) is the most powerful non-invasive functional imaging technique. It has the potential to radically revolutionize researchers’ understanding of the physical basis of the brain and provide valuable tools for clinical and research purposes [14]. Because the neurological and behavioral effects of acute drug administration are often of short duration, the temporal pattern of change over a short time is critical. Such dynamic alterations can be detected by fMRI, which can reflect average values over shorter periods. fMRI studies reveal a complex dynamic pattern of brain changes during drug intoxication, with different temporal patterns, with some regions activated and others blocked. Functional connectivity in brain networks is commonly generated by analyzing fMRI time series, and functional brain networks characterize the statistical correlation patterns between neuronal regions. In the last decade, the significant progress has been made in using fMRI data for brain functional network analysis [15]. Alterations in functional connectivity between brain regions have been extensively studied in the field of brain disorders, as well as the association between cognitive impairment [16] and degenerative neurological and psychiatric disorders [17].

## Related work

Machine learning techniques have been widely applied in recognition of medical scenes [18]. In brain image computing, machine learning-based artificial intelligence approaches powerful capabilities to drive brain image analysis technology forward [19, 20], effectively refining physicians’ diagnoses and improving the accuracy of disease prediction. Recent advances in machine learning, particularly in deep learning, contribute to identifying, classifying and quantifying brain images [21]. Deep learning-based brain image analysis methods [22, 23] for brain disease research can explore the disease’s mechanism and understand the brain disorder process. The core of these advances is the capability to automatically generalize hierarchical features [24] from data rather than manually discovering and designing features [25] depending on specific knowledge.

Because of the improvement of deep learning, the performance of several neuroscience applications has concurrently increased dramatically [26]. Deep learning techniques are a novel and efficient way of processing and extracting low-dimensional information from high-dimensional brain imaging data. For example, convolutional neural network (CNN) approaches reduce medical image data dimensionality to identify patterns in brain imaging [27]; generative adversarial networks (GAN) methods [28, 29] are frequently employed in medical image fields, which are built on variational inference methods. Generative adversarial techniques can simulate the actual distribution of data to decrease noise interference and improve model resilience [30].

For the past few years, deep learning medical image analysis methods based on graph neural networks have yielded successful results in the fields of disease classification and marker detection [31]. Graph neural networks are learning models that combine attributes and structural features into a single graph for processing. In contrast to traditional graph methods, graph neural networks can automatically propagate information carried by neighboring nodes, which can then be used to analyze patterns of brain disorders.

However, processing network-structured data to obtain interpretable and determinable biomarkers is still challenging by existing methods. Traditional statistical-based methods require complex and redundant computational operations on image data. In contrast, by adopting common deep learning methods, the high dimensionality and small sample size of fMRI image data lead to difficult training, and complex features result in low identification accuracy. To address these issues, we develop a novel learning framework with feature selection techniques and make the following contributions:

1. Spatial attention recurrent network (SARN) is designed to identify effective patterns of addiction-related brain networks from fMRI data, which can learn the spatial structure and sequential information.
2. A Bayesian feature selection approach is utilized to obtain effective and interpretable brain network embeddings for better identification performance.
3. The feature-selected brain regions can be considered addiction-related biomarkers verified by our experiments and neuroscience knowledge. And the discovery of these brain regions will help research addiction mechanisms.

This work extends a preliminary version of the paper presented at the 2022 International Conference on Brain Informatics [32]. We build on the work of that paper by supplementing analyses and expanding the encoder with a novel recurrent network that can better handle the temporal information of dynamic brain networks.

**Fig. 1:**
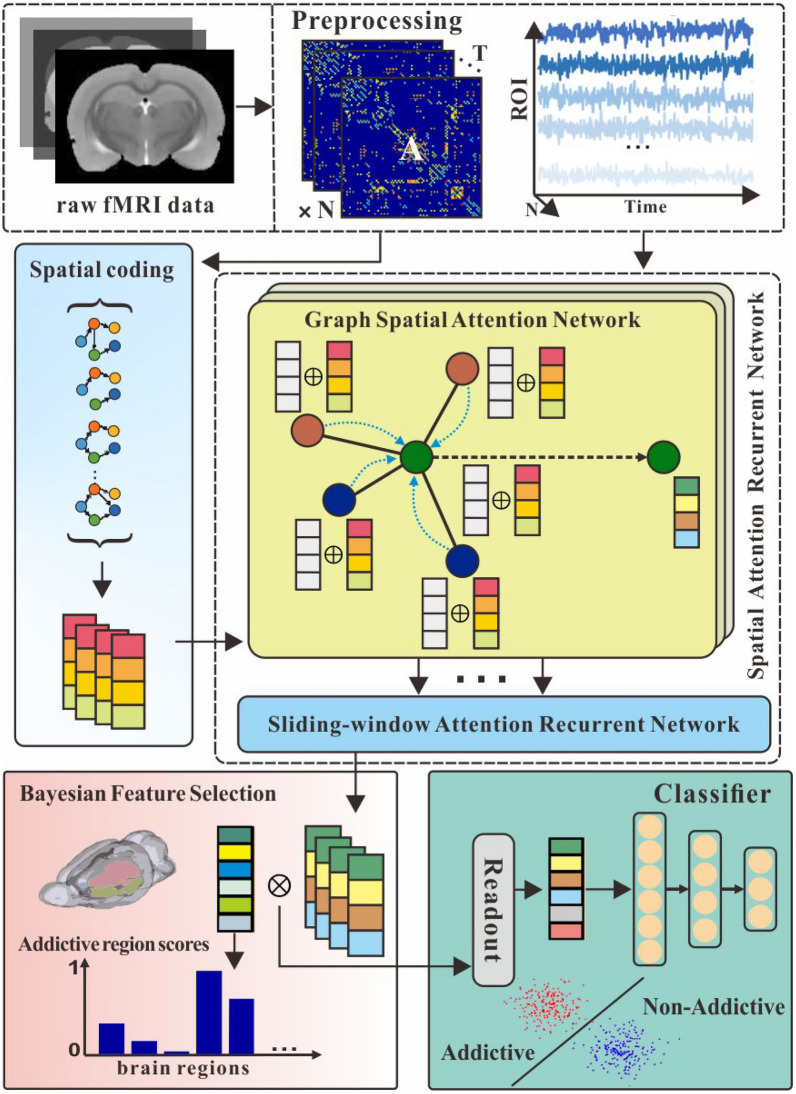
Proposed spatial attention recurrent network with Bayesian feature selection for identifying brain addiction. The top part of the figure is the raw fMRI preprocessing and brain network construction, the middle is the designed encoder, and the bottom is the Bayesian feature selector and addiction classifier

## Method

As shown in Fig. 1, the detailed architecture of the proposed framework is demonstrated. Our framework comprises three main components: 1) SARN encoder consists of graph positional attention layers and sliding-window attention recurrent layers; 2) a feature selector with Bayesian feature selection strategy; and 3) a classifier for identifying addiction-related brain network embeddings.

Raw fMRI data are firstly fed as input and preprocessed into network data with structural information and attributes. Generally, in the encoder(*E*), self-attention mechanism is adopted to transform the time series of brain regions $X=\left\{ x_n\right\} ^N_{n=1}\in {\mathbb {R}}^{N\times D}$ and dynamic brain functional connections $\left\{ A^t\right\} ^T_{t=1}$ into the embeddings $Z=\left\{ z_n\right\} ^N_{n=1}\in {\mathbb {R}}^{N\times d}$. Moreover, in the feature selector, the latent binary random vectors $B=\{b_n\}^N_{n=1}$ are created to infer the posterior probability distribution and select more efficient brain regional features. Therefore, the encoder is trained with double objectives: a Bayesian feature selection loss considered as the feature sparsity penalty and a classification loss for identifying nicotine addiction. After sufficient training, the model in the framework can finally output addiction probability scores of specific brain regions and addiction brain network identification results.

### Graph spatial attention network

The graph spatial attention encoder aims to embed the regional brain imaging features aggregated with dynamic brain network attributes into a low-dimensional latent space. The proposed layer that composes the encoder is based on the graph attention networks (GAT) [33] with the addition of spatial encoding. It allows each regional brain node to focus adaptively on other nodes according to the spatial information of the graph-structure connectivities in the brain networks.

Therefore, the attention coefficient, which is combined a shared attentional mechanism and spatial encoding for brain connectivities, can be expressed as:

1 $$\begin{aligned} &\mathbf {\alpha }^{l}_{(i, j)}= \\&\quad \frac{\exp \left( \tanh \left( \left[ {\textbf{h}}_{(i)}^{l} {\textbf{W}}^{l} , {\textbf{h}}_{(j)}^{l} {\textbf{W}}^{l}\right] \cdot {\textbf{c}}^{l}+s_{\psi (x_i,x_j)}\right) \right) }{\sum _{j \in {\mathcal {N}}(i)} \exp \left( \tanh \left( \left[ {\textbf{h}}_{(i)}^{l} {\textbf{W}}^{l} , {\textbf{h}}_{(j)n}^{l} {\textbf{W}}^{l}\right] \cdot {\textbf{c}}^{l}+s_{\psi (x_i,x_j)}\right) \right) }, \end{aligned}$$

where $h^l_{(i)}$ is a hidden representation for brain node *i* at *l*th layer, $W^l\in {\mathbb {R}}^{d_l\times d_{l+1}}$ is a parameterized weight matrix considered as the graph convolutional filter, $c^l$ is a weight vector that can be learned in the train phase, and $S_{\psi (x_i,x_j)}$ is a scalar that can be learned and is indexed by $\psi (x_i,x_j)$ with positional information. It indicates the spatial encoding and is accessible throughout all layers.

Formally, let ${\textbf{h}}_{(i)}^{l+1}$ represent the output representation at *l*th layer, our graph spatial attention layer is given as follows:

2 $$\begin{aligned} {\textbf{h}}_{(i)}^{l+1}=\sigma \left( \sum _{j \in {\mathcal {N}}(i)} \mathbf {\alpha }_{(i, j)}^{l} {\textbf{h}}_{(j)}^{l} {\textbf{W}}^{l}\right) . \end{aligned}$$

In Eq. 2, the feature propagation mechanism aggregates the effects across overall neighboring brain nodes and attaches spatial encoding information from dynamic brain network connectivity $\left\{ A^t\right\} ^T_{t=1}$.

**Fig. 2:**
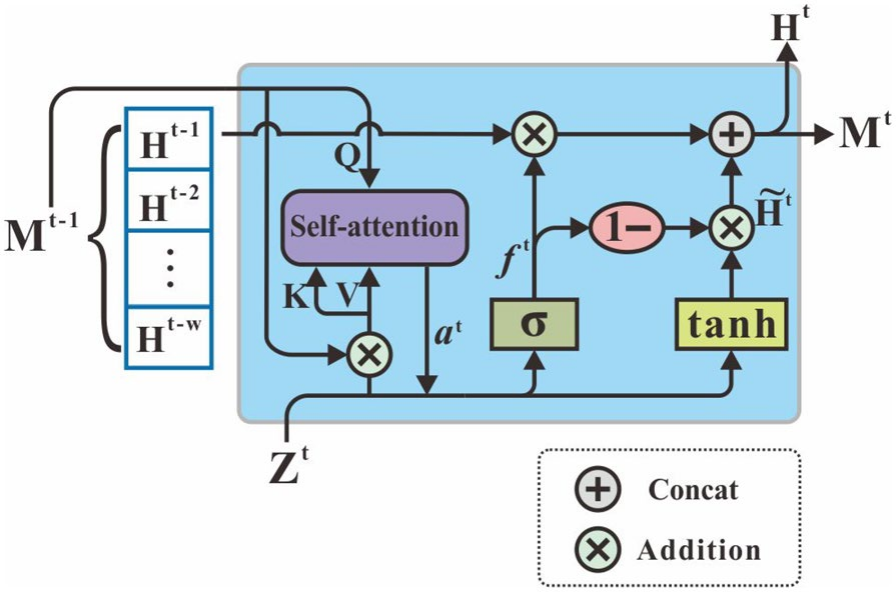
The detailed unit of sliding-window attention recurrent network

### Sliding-window attention recurrent network

As is known, the sliding-window technique [34] is commonly used for capturing dynamic changes of functional brain imaging and extracting efficient time courses. Inspired by this basic approach and attention mechanism [35], sliding-window attention network is designed for further processing network embeddings and extracting the time sequential representation of dynamic functional attributions. Sliding-window attention recurrent layer consists of individual units in series; the structural details of the unit are shown in Fig. 2. In this network, the data input is the brain network embedding from the former layer at different time steps, and the output is the brain network representation of the whole time series. We consider previous memory state $M^{t-1}=[H^{t-1},H^{t-2},...,H^{t-w}]$ as the time window interest of the dynamic brain networks within *w* time steps. At step *t*, the output embeddings of graph spatial attention network $Z^t$ become the original input of this recurrent unit, and the input query matrix of self-attention $Q=Z^t$, key and value matrices are $K,V=[Z^t,M^{t-1}]$. To implement self-attentions, the weighted coefficients $e^t_m$ and attention coefficients $a^t_m$ are calculated as given below:

3 $$\begin{aligned} e^t_m= & {} \frac{W_qQ \cdot (W_kK_m)^T}{\sqrt{d_k}}, \end{aligned}$$

4 $$\begin{aligned} a^t_m= \,& {} SelfAttn(Z^t,[Z^t,M^{t-1}],[Z^t,M^{t-1}]) \nonumber \\=\, & {} \frac{exp(e^t_m)}{\sum ^{w}_{m=0}exp(e^t_m)}. \end{aligned}$$

The intermediate hidden state $H^{t-1}$ is updated as $H^t$, and $M^t$ is updated by adding $H^T$ to $M^{t-1}$. The calculation that occurs during the update is shown in the following equations:

5 $$\begin{aligned} f^t= & {} \sigma (W_fZ^t+U_fa^tV+b_f), \end{aligned}$$

6 $$\begin{aligned} {\tilde{H}}^t= & {} tanh(W_hZ^t+U_ha^tV+b_h), \end{aligned}$$

7 $$\begin{aligned} H^t= & {} f^t\odot H^{t-1}+(1-f^t)\odot {\tilde{H}}^t. \end{aligned}$$

We finally get $H^t$ containing the spatial and temporal features after this network. Although it is similar to simplified LSTM [36] or GRU with attention [37], the differences are that it only retains the forget gate to reduce the redundancy of the model and combines sliding-window and self-attention mechanisms to obtain better temporal features under the time window.

### Bayesian feature selector

To find the most effective features for identification from many regional brain features and to acquire a set of fewer but discriminative biomarkers to reduce classification error, we employ the Bayesian feature selector. We define ${\textbf{H}}=\{H_1^o,...,H_n^o\}$ and ${\textbf{Y}}=\{y_1,...,y_n\}$ as the output features from the encoder and labels of addiction or not. By introducing binary masking matrix *B* to achieve the goal of selecting features, the expected posterior distribution is denoted as $p({\textbf{B}}\mid {\textbf{H}},{\textbf{Y}})$ and an approximate distribution is represented as $q(\cdot )$. In order to improve the identification performance and the accuracy of the model in discriminating features, in the view of variational method [38] and Bayesian inference, we optimize the model by minimizing the KL divergence between the posterior distribution and the approximate distribution:

8 $$\begin{aligned}&\underset{q(\cdot )}{{\text {argmin}}}KL(q({\textbf{B}})\Vert p({\textbf{B}}\mid {\textbf{H}},{\textbf{Y}}))=\\&\quad -E_{q}\left[ \log \left( p({\textbf{B}}\mid {\textbf{H}},{\textbf{Y}})\right) \right] +K L\left( q\left( {\textbf{B}}\right) \Vert p\left( {\textbf{B}}\right) \right).\end{aligned}$$

In Eq. 8, the first term corresponds to a binary cross-entropy loss for identification task where the input features ${\textbf{H}}$ are masked by ${\textbf{B}}$, and the second term becomes a loss for learning the probability scores ${\textbf{z}}$ which is used to compute the binary matrix ${\textbf{B}}$ by Bernoulli sampling method:

9 $$\begin{aligned} {\textbf{b}}_{n}=\sigma \left( \frac{\log \left( {\textbf{z}}\right) -\log \left( 1-{\textbf{z}}\right) +\log \left( {\textbf{u}}_{n}\right) -\log \left( 1-{\textbf{u}}_{n}\right) }{r}\right) , \end{aligned}$$

where ${\textbf{u}}_n$ is sampled from a uniform distribution from 0 to 1, and *r* is the relaxation parameter of Bernoulli sampling.

### Classifier and loss function

To integrate the information of each node for the graph-level identification, we utilize a readout function to cluster node features together by simply averaging them:

10 $$\begin{aligned} {\mathcal {R}}({\textbf{H}})=\sigma \left( \frac{1}{N} \sum _{i=1}^{N} \textbf{h}_{i}\right) , \end{aligned}$$

where $\sigma$ is nonlinear activation function. The readout function is similar to the graph pooling operation. Other graph pooling methods can be used to replace it. The selected and readout features are delivered to a multi-layer perceptron (MLP) to derive the final identification of predicted labels ${\hat{y}}$.

The total loss function is the interpretation of Eq. 8:

11 $$\begin{aligned} {\mathcal {L}}\left( {\textbf{X}}, {\textbf{A}}\right)&= -\sum _{n=1}^{N}\left( y_{n} \log \left( {\hat{y}}_{n}\right) +\left( 1-y_{n}\right) \log \left( 1-{\hat{y}}_{n}\right) \right) \\&\quad +K L\left( {\text {Ber}}\left( {\textbf{z}}\right) \Vert {\text {Ber}}({\textbf{s}})\right) . \end{aligned}$$

The first term is used to guide the MLP in the classification of the selected features. Furthermore, the second term is applied for training the selector to learn the probability mapping to the feature mask. Here, ${\text {Ber}}({\textbf{s}})$ is a binary random vector that contains sparse elements for the purpose of complying with sparsity.

## Experiments

In this section, we first evaluate the capability of the proposed framework in identification performance through an ablation study and comparison experiment. And we utilize four kinds of binary classification metrics to measure the experiment results of dynamic brain network identification. Then, we analyze the scores of the other framework output to detect addiction-related brain regions. These identified brain regions are visualized and validated as interpretable biomarkers.

**Fig. 3:**
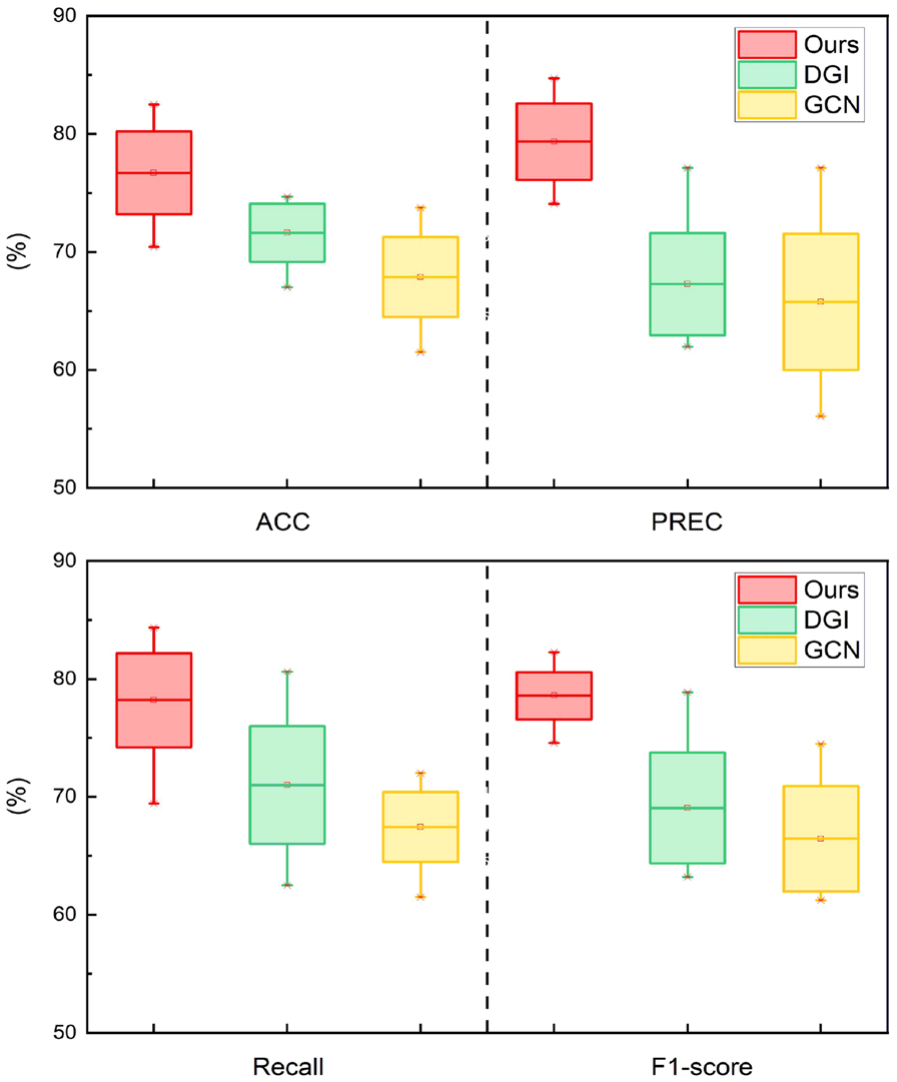
Identification experiments on comparative methods. The red box represents our method, and the green and the yellow are DGI and GCN methods. The maximum, minimum, mean, and quartiles are shown in the box plot

**Table 1:** Ablation study for assessing the efficiency of the encoder and feature selection

Encoder	Feature selection	Metrics			
		ACC	PREC	Recall	F1-score
GAT	$\checkmark$	67.75	73.33	72.67	73.00
SARN	$\times$	75.50	77.83	80.25	79.02
SARN	$\checkmark$	**81**.**25**	**79**.**50**	**83**.**33**	**81**.**37**

The bold values indicate that the best performance is obtained by SARN with feature selection

**Fig. 4:**
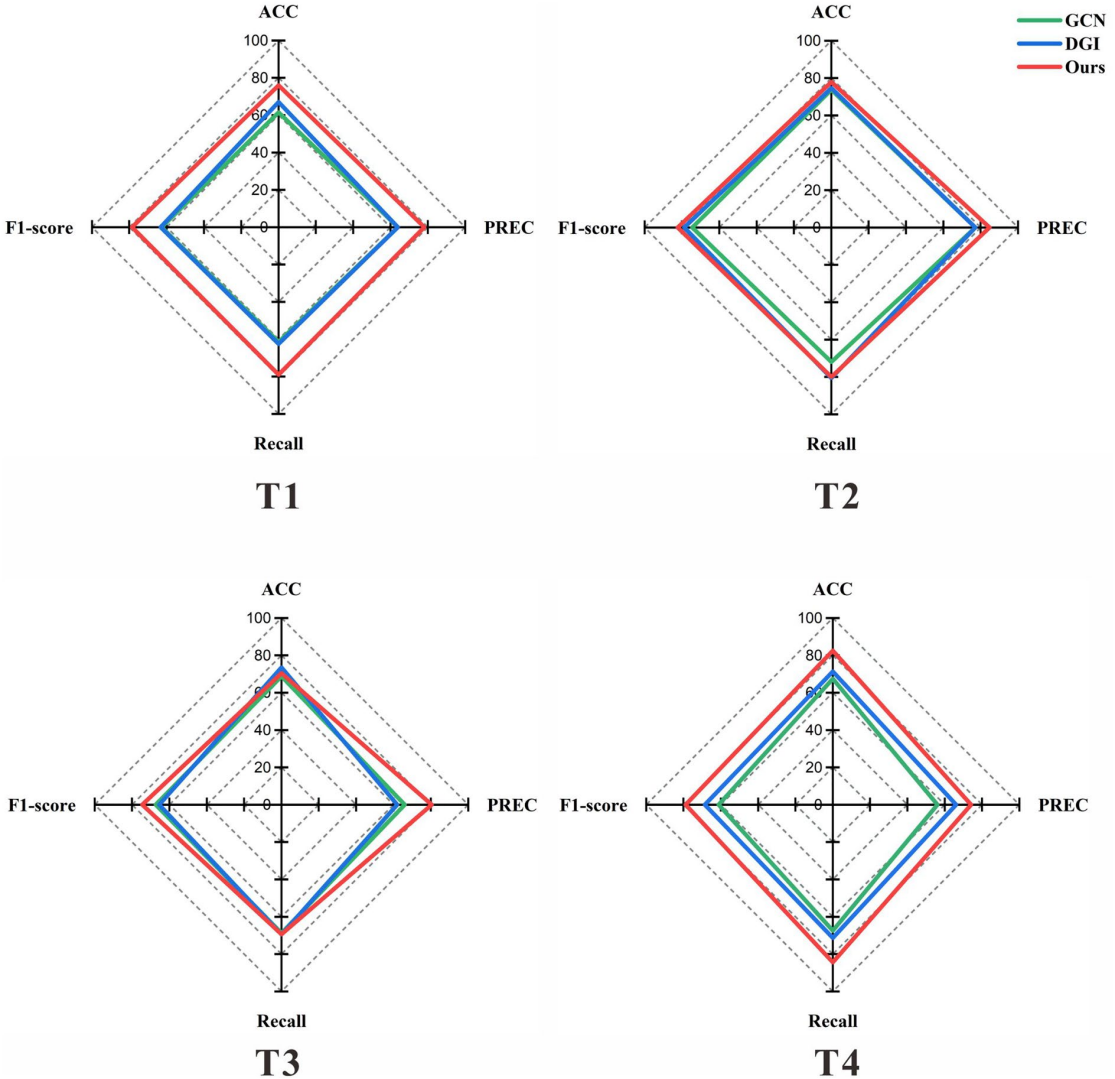
Comparison of identification results at different four time steps

**Fig. 5:**
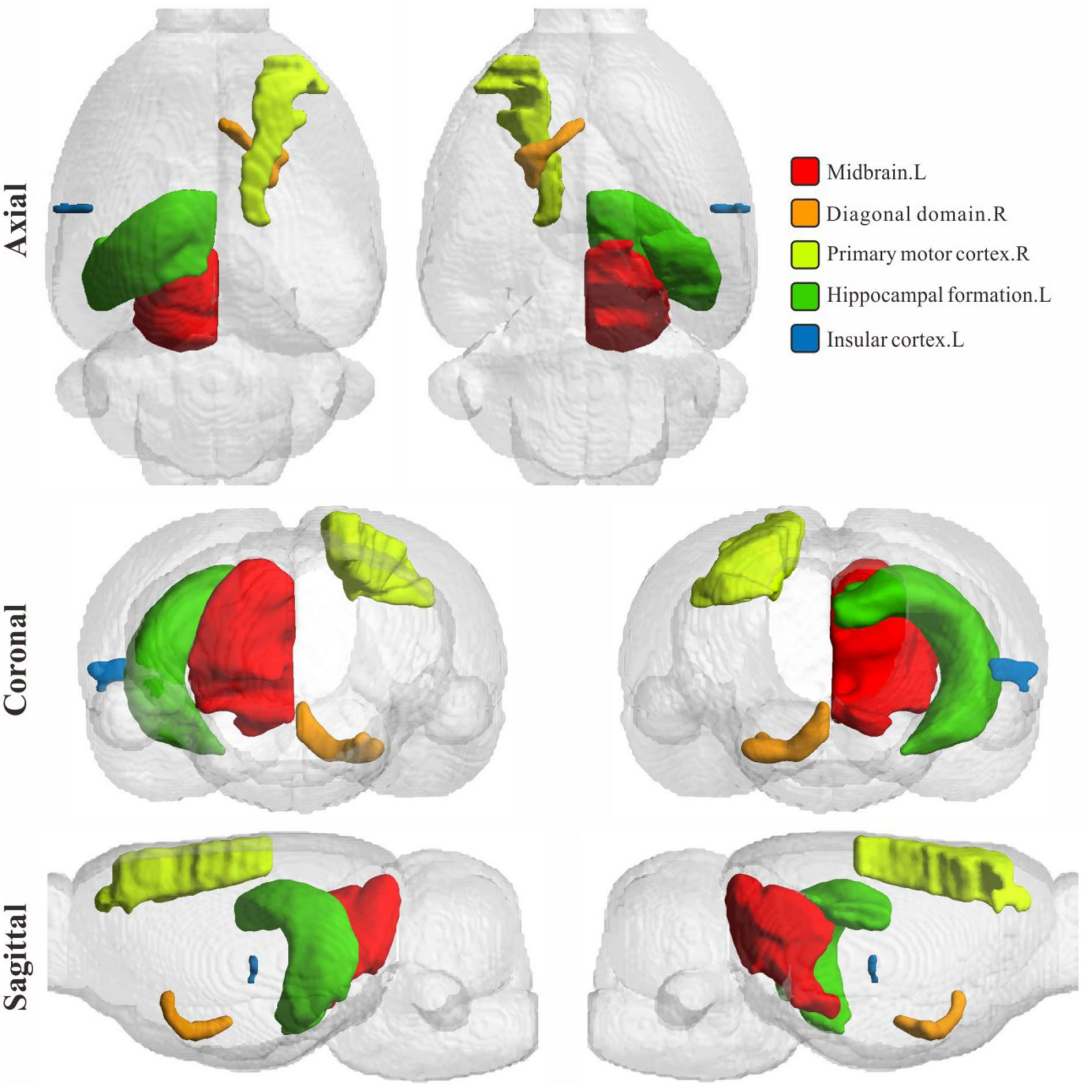
Visualization of top five addiction-related brain regions with the highest BFS-weighted scores

### Dataset and preparation

An fMRI dataset on the addiction animal model is adopted for our experiments. It contains 8 normal control fMRI, addiction-irrelevant samples, and 16 addiction-related fMRI samples, each of which has 800 time points. The quality of these image data is strictly controlled, with the signal-to-noise ratio as large as possible. To transform fMRI data into the dynamic brain network data, the following preprocessing is implemented. Functional data are aligned and unwarped to account for head motion, and the mean motion-corrected imaging is coregistered with the higher resolution anatomical T2 imaging. Then the preprocessing images are smoothed by an isotropic Gaussian kernel with a 3-mm full-width at half-maximum. With the Wistar rat brain atlas [39], 150 rat brain regions are defined and fixed in the normalized space. We assessed the functional connection between regional time series by calculating the Pearson correlation coefficient, resulting in a $150\times 150$ adjacent matrix for each time step, and we divided the whole time series, 800 time points, into 4 time steps equally. The adjacency matrix and the temporal properties of brain regions at all time steps form the dynamic brain network data as the input dataset. Besides, the major part of the preprocessing steps is done with the assistance of toolboxes, including Statistical Parametric Mapping 12 (SPM12) [40] and Graph Theoretical Network Analysis (GRETNA) [41].

### Implementation detail

The PyTorch backend was used to implement FGSAN. One Nvidia GeForce RTX 3080 Ti was used to speed up the network’s training. During training, the learning rate was set at 0.001, and the training epoch was set to 1000. Adam was used as an optimizer with a weight decay of 0.01 to reduce overfitting. The proposed SARN encoder is composed of three graph spatial attention layers and one sliding-window attention recurrent layer. All trials are repeated ten times, and the results are averaged. The regularization value was set to 0.5 for all datasets and techniques.

### Metrics

Evaluation of binary classification performance is based on quantitative measures in four key metrics: (1) accuracy (ACC); (2) precision (PREC); (3) recall; and (4) F1-score. Our proposed method is evaluated by fourfold cross-validation.

### Ablation study of identification performance

As indicated in Table 1, we conducted ablation research on identification to evaluate the effectiveness of our proposed encoder and Bayesian feature selector, and two significant results are achieved:

1) In the comparison to the baseline encoder, GAT showed impressive performance on the binary addiction-related classification. This is due to the fact that the spatial encoding enables the self-attention mechanism to get more positional information and achieve better graph embeddings, and the recurrent network learns more effective representations in the temporal dimension;

2) The Bayesian feature selector comprehensively improves performance of the identification methods. It represents that feature selection plays its role as an auxiliary to identifying the graph-structure patterns, and task-involved embeddings are selected to make the model perform better on the classification.

**Table 2:** TOP five regional brain biomarkers extracted by the FGSAN model.

No.	ROI name of biomarkers	References
1	Midbrain.L	[42]
2	Diagonal domain.R	[43]
3	Primary motor cortex.R	[44]
4	Hippocampal formation.L	[45]
5	Insular cortex.L	[46]

**Fig. 6:**
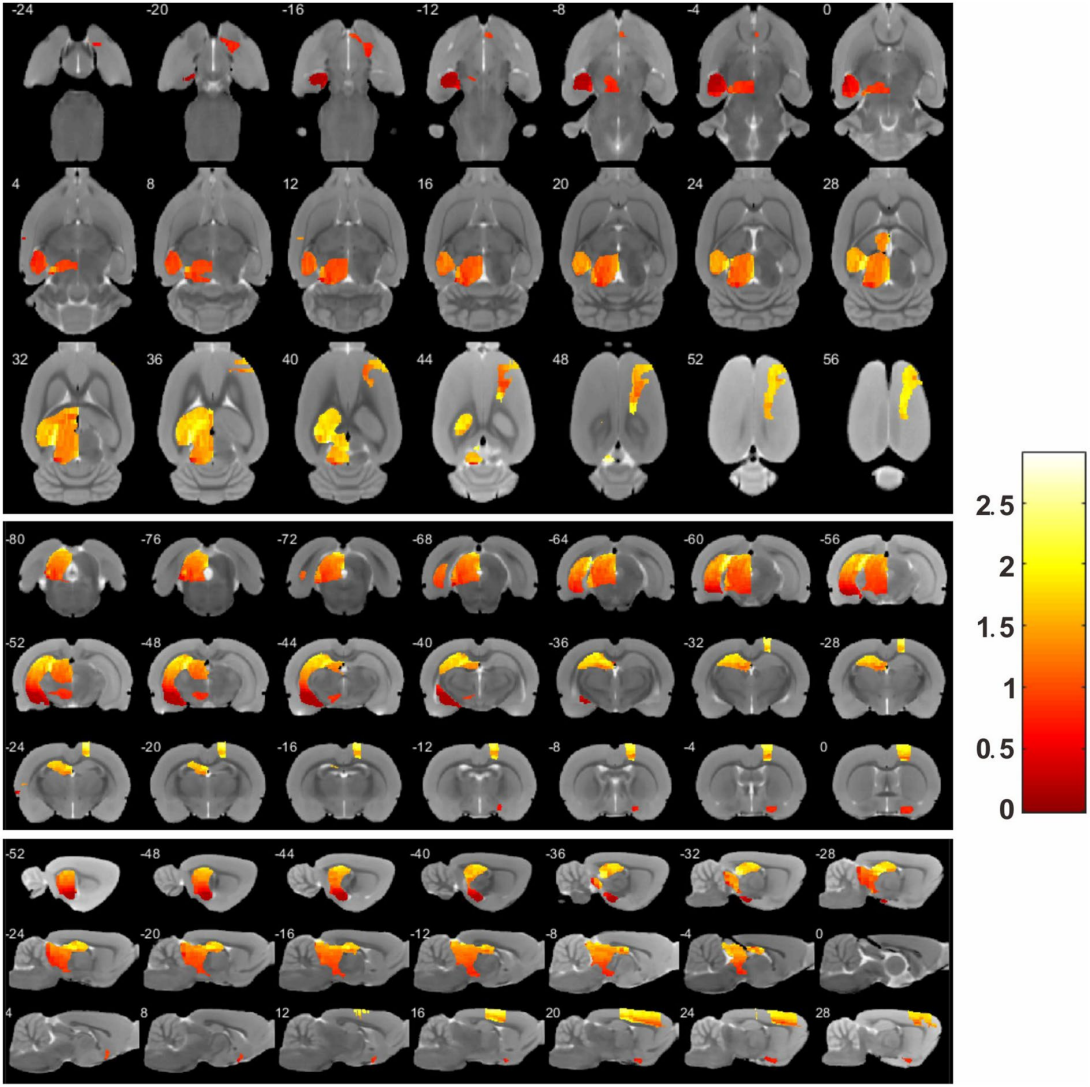
BOLD signal visualization of addiction-related brain regions in original imaging data

### Comparison of identification performance

We conduct comparative experiments to verify the superiority of the method proposed in this part of the section. It is compared with the methods with graph neural networks, including GCN [47] and DGI [48]. GCN is a classical graph learning model for the graph-level classification task. Based on GCN, DGI learns node embeddings in an unsupervised technique. And it can continuously optimize model results by maximizing the mutual information between the local representation and the global representation. After our experimental verifications, it is found that the method of our framework is significantly better than contrastive methods on classification metrics. As shown in Fig. 3, SARN with BFS outperforms compared with DGI and GCN in every indicator of the binary classification experiment. It is probable that our approach has a stronger capacity to identify patterns of addiction in the brain. Moreover, in order to explore the identification of the dynamic temporal properties, we perform different classification experiments according to the four divided time steps. And the results are shown in Fig. 4, where it can be observed that our model shows robustness and superior performance in the different time steps.

### Interpretable brain regional biomarkers

The brain regional features that are selected by our method have the corresponding selection probability frequency and scores, which are presented importance of brain regions and criteria for inferences. We weight these values cumulatively to make the statistics. As shown in Table 2, the five brain regions with the highest weights are Midbrain.R, Diagonal domain.R, Primary motor cortex.R, Hippocampal formation.L, and Insular cortex.L. The higher probability score not only means that the brain region is more deterministic in inferences, but also means that this feature is more implied to the differences caused by addiction, and the corresponding brain region is more addiction-related. To validate these addiction-related brain regions are interpretable, we confirmed them in terms of neuroscience-supported knowledge and imaging.

On one side, these five brain regions discovered by our method have been proven to be associated with nicotine addiction in previous research work. We collect and categorize the relevant references of prior research on each brain region and also list them in Table 2. In addition, we visualized the locations of these five brain regions. As shown in Fig. 5, the locations of the five addiction-related brain regions found by the model in the rat brain are shown in axial, coronal, and sagittal directions.

On the other side, we observe the BOLD signal of the discovered addiction-related brain regions in the raw images, which indicates the functional activity of the brain, and find that the top five brain regions have significant BOLD signal differences between the addiction and non-addiction animal models. In the same way, we visualize these regions, respectively, in Fig. 6. It is verified as evidence for becoming the interpretable biomarkers from the raw image data.

## Conclusion

In this research, we propose a new framework, spatial attention recurrent network (SARN) with Bayesian feature selection, for discovering effective and interpretable regional brain biomarkers and utilizing features of these biomarkers to identify the addiction-related brain network patterns dynamically. Our model is investigated and discussed in detail through designed experiments to present the superiority of the encoder and feature selector in the proposed framework. We obtain better results than the comparative method by using the selected graph representations for classification, indicating an advantage in graph feature extraction that may yield better graph embeddings in the latent space. More significantly, the importance of these regional features can be well explained in the neuroscience of addiction, and the direct support of the corresponding biomarkers can be found in the original image data. Delving into these addiction-related brain regions and matching the interconnections between them to the different kinds of addiction mechanisms and studying such causal addiction circuits and mechanisms will be the direction of our continuing research in the future.

## Data Availability

Data used in the manuscript will be available on request after the manuscript is published.
